# The use of nocturnal flights for barrier crossing in a diurnally migrating songbird

**DOI:** 10.1186/s40462-021-00257-7

**Published:** 2021-04-26

**Authors:** Christie D. Lavallée, Saeedeh Bani Assadi, Alicia M. Korpach, James D. Ray, Jason D. Fischer, Joe Siegrist, Kevin C. Fraser

**Affiliations:** 1 Department of Biological Sciences, University of Manitoba, Winnipeg, MB Canada; 2 Consolidated Nuclear Security, LLC, Pantex Plant, Amarillo, TX 79120 USA; 3 Disney’s Animals, Science, and Environment, Lake Buena Vista, FL 32830 USA; 4 Purple Martin Conservation Association, Erie, PA USA

**Keywords:** Circadian timing, Aerial insectivores, GPS tracking, Individual plasticity, Ecological barrier, Fly-and-forage migration, Diurnal migrant

## Abstract

**Background:**

The migration patterns of land birds can generally be divided into those species that migrate principally during the day and those that migrate during the night. Some species may show individual plasticity in the use of day or night flight, particularly when crossing large, open-water or desert barriers. However, individual plasticity in circadian patterns of migratory flights in diurnally migrating songbirds has never been investigated.

**Methods:**

We used high precision GPS tracking of a diurnal, migratory swallow, the purple martin (*Progne subis*), to determine whether individuals were flexible in their spring migration strategies to include some night flight, particularly at barrier crossing.

**Results:**

Most (91%) of individuals made large (sometimes > 1000 km), open-water crossings of the Caribbean Sea and the Gulf of Mexico that included the use of night flight. 32% of all water crossings were initiated at night, demonstrating that night flight is not only used to complete large crossings but may confer other advantages for diurnal birds. Birds were not more likely to initiate crossings with supportive winds, however crossings were more likely when they reduced travel distances. Our results are consistent with diurnal birds using night flight to help achieve time- and energy-savings through ‘short cuts’ at barrier crossings, at times and locations when foraging opportunities are not available.

**Conclusions:**

Overall, our results demonstrate the use of nocturnal flight and a high degree of individual plasticity in migration strategies on a circadian scale in a species generally considered to be a diurnal migrant. Nocturnal flights at barrier crossing may provide time and energy savings where foraging opportunities are low in an otherwise diurnal strategy. Future research should target how diel foraging and refueling strategies support nocturnal flights and barrier crossing in this and other diurnal species.

**Supplementary Information:**

The online version contains supplementary material available at 10.1186/s40462-021-00257-7.

## Background

The migratory movements of animals can often be characterized by whether they occur primarily during daylight hours or during the night. Many diurnal land bird species that are usually active only during the day, migrate during the night. This can confer advantages of time and energy savings, and can reduce predation risk [1, 2]. However, these mainly nocturnal migrants can also sometimes be observed moving in a migratory direction during the day [1], demonstrating some flexibility in circadian timing strategies. In many cases, these movements can be attributed to completing the crossing of a major ecological barrier, to re-orientate and correct movement errors, or to avoid poor weather [1, 3–5]. For example, recent tracking data has shown that nocturnally migrating songbirds crossing the Sahara Desert during spring migration use some day flight in order to complete the crossing of this inhospitable barrier [2–4]. For other migratory land birds, day-night divides may be more impermeable barriers to migratory activity, or alternatively, flight pattern structure may be maintained despite changes in light regimes. For example, some nocturnal species, such as nightjars, may be restricted to nocturnal migration throughout their journeys [6, 7] and some diurnal migrants moving north of the arctic circle may maintain the timing and duration of their diurnal flights, even when there are longer hours of daylight available [8].

Diurnally migrating birds need to both migrate and forage during the daytime and may adopt a fly-and-forage strategy, where they both migrate and refuel during the day [9, 10]. A fly-and-forage strategy may be supported by foraging opportunities that may occur during flight (such as for aerial insectivores), or at pauses during, or after, daily migratory flights [4, 10]. It is predicted that diurnal migrants may incorporate nocturnal flights when they cannot benefit from energy deposition during a fly-and-forage strategy, such as when crossing ecological barriers or habitats with sub-optimal foraging [4]. Diurnal Eleonora’s falcons (*Falco eleonorae*) were found to be flexible in their use of day or night flights during migration, particularly when crossing ecological barriers that did not provide insect rich areas for foraging [11]. However, whether diurnally migrating songbirds presumed to use a fly-and-forage strategy of migration, such as swallows, use nocturnal flight during migration has been little investigated [12]. Recent investigations of swallow diurnal versus nocturnal movements confirmed only daytime movements [12]. Recent migration tracking of some diurnally migrating species at large barrier crossings suggest some night flight may be incorporated, because duration of crossing seemed to exceed daylight hours [13, 14]. However, this has not been directly examined.

The diversity of migration timing strategies, especially in ecological barrier crossing, can also reflect species-specific, or intraspecific strategies [4, 5]. For example, whinchats (*Saxicola rubetra*) crossing the Sahara Desert had higher predicted migration speeds as compared to when crossing the Mediterranean Sea, which is a considerably narrower barrier [15]. Investigations of the flexibility of diurnal or nocturnal migration over ecological barriers (water or land) and across full migratory routes remain rare. New, high spatio-temporal precision in tracking technology that can be applied to studies of even small (< 100 g) migrants offers new opportunities to investigate migration timing strategies on a circadian scale.

As a trans-hemispheric long-distance migratory swallow, purple martins (*Progne subis*) are thought to be exclusively diurnal birds that feed and migrate during the day using a fly-and-forage strategy [16]. They migrate up to 10,000 km seasonally between North American breeding sites and South American overwintering sites choosing migratory routes that cross over the Gulf of Mexico or the Caribbean Sea, suggesting some night flight may be used to complete the crossings [13, 14]. Our aim in this study was to apply high precision tracking to determine whether this diurnal songbird uses both day and night flight to accomplish large ocean barrier crossings during spring migration, and/or whether they use nocturnal flights generally during their migrations. Through this investigation, we tested the hypotheses that, 1) these diurnal migrants may incorporate night flight at barrier crossing where foraging opportunities are suboptimal, and 2) that night flights are associated with advantages of facilitating winds and/or a reduction of migration distance as a component of time- or energy-saving strategies [4].

## Methods

During the 2017 to 2019 breeding season, we deployed a total of 98 GPS units (Pinpoint 10, Lotek Inc.) on adult purple martins at four North American breeding locations (supplemental material, Table A). Purple martins were captured using drop-door traps at their nest boxes. GPS units were mounted onto adults using a leg-loop backpack harness made of Teflon ribbon [17]. The mass of tag (1.5 g) and harness was less than 3% of an adult purple martin’s body mass.

Tags were pre-programmed to collect positional fixes across spring migration (January to April) at prescribed times that enabled the partitioning of day versus night flights. We programmed tags to align with breeding population-specific timing (FL: January, TX: March, MB: April; Table 1), previously identified through the use of light-level geolocators ([13, 14], Fraser et al. unpub. data), to capture the spring migration routes and timing we required for this investigation. Tags were programmed to detect and save locations two or three times a day: 0600 and 1800 h Central Daylight Time (CDT) (*n* = 8, Manitoba (MB) and Texas (TX) colonies); 0400 and 1600 h Eastern Daylight Time (EDT) (*n* = 2, Pennsylvania (PA) and Florida (FL)); 0400, 1000, and 1600 h (CDT) (*n* = 1,TX); 0000, 0600 and 1800 h (CDT) (*n* = 1,TX). Detections at 0000-h and at 1800 h both reflected a portion of nocturnal flight [7], and therefore were combined to create a 12-h night flight interval to make data comparable to those from other tags. Similarly, detections at 1000 h were combined with detections at 0400 h to make a 12-h day flight interval for better comparison with other tags.

**Table 1:** Location, timing, and other migration track details of each individual purple martin used in this study. A total of their day and night flights over water was calculated using GPS positions logged every 12 h

Bird ID	Sex	Breedingground	Winteringlocation (lat., long.)	Migrationtrack start	Migrationtrack end	Number of dayflights over water	Number of nightflights over water
1598	M	Florida	−2.31, − 54.12	26/01/2018	NA	1	2
1602	M	Florida	−1.15, −62.09	14/01/2018	5/02/2018	3	2
2810	F	Texas	−1.37, −61.70	08/03/2020	NA	1	2
48041	M	Texas	7.84, −69.95	12/03/2018	15/04/2018	2	2
48042	F	Texas	NA	12/03/2018	22/03/2018	1	1
48045	F	Texas	−3.78, −58.3	27/03/2018	17/04/2018	2	2
48046	M	Texas	−2.83, −60.51	30/03/2018	20/04/2018	3	3
48051	F	Texas	−6.84, −51.7	14/03/2018	16/04/2018	1	–
48052	M	Texas	−2.12, −55.56	17/03/2018	4/04/2018	3	2
48794	M	Texas	16.06, −89.12	12/03/2019	29/03/2019	1	1
2177	M	Manitoba	2.36, −65.23	18/04/2019	07/05/2019	3	2

GPS units were retrieved in the year following deployment using the same methods of capture. PinPoint Host software (Lotek Inc.) was used for data extraction. We defined flights between 1800 to 0600 and 1600 to 0400 h as nocturnal flight and those between 0600 to 1800 and 0400 to 1600 as diurnal flight. Migration distances were measured using Google Earth [18] and as geodesic distances (km) between fixes using the R package *geosphere* [19].

Because the GPS tags collected locations on fixed schedules, the amount of daylight that occurred during the tracking periods varied as the season progressed and birds moved substantial distances. We determined the amount of available daylight in each 12 h track segment to address the possibility that some daylight was present during the ‘nighttime’ flight segments. Daylight hours are defined as the time between sunrise and sunset (R package *suncalc* [20]), and the amount of daylight per track was calculated according to the GPS locations and fix times at the beginning and end of a 12 h track segment (i.e. time between sunrise or sunset at the bird’s start location and sunset or sunrise at the bird’s end location). Log-transformed distances were regressed against amount of daylight in the 12 h segments in a linear model (*n* = 190 day segments, 203 night segments).

### Barrier-crossing model

We tested the effect of wind assistance and potential distance savings on the decision to cross a waterbody or detour around it. We used the last GPS location recorded for each individual before their track either continued across a waterbody (*n* = 22), or reoriented to circumnavigate the waterbody (*n* = 6), and classified those departure points into binary categories of ‘cross’ or ‘detour’.

#### Distance savings (water:land) covariate

We compared the distances of water crossings to circumnavigations around those waterbodies, which represent the alternatives that a bird would have when faced with a decision at the coast to launch a water crossing or reorient to remain over land. For each track that crossed a waterbody, we measured the corresponding hypothetical circumnavigational route distance, using the minimum number of connecting lines required to constrain the path over land. Conversely, where a bird had actually circumnavigated a waterbody, we measured the hypothetical water crossing distance to represent the alternative route. Here, we measured the geodesic distance between the GPS location at the departure point (the point at which the bird had made the decision to reorient) and the next closest GPS location on the far side of the waterbody. This GPS location was always a point after which the bird was moving away from the waterbody, in a northbound migration direction. For each individual at each waterbody, we divided the water crossing distance by the circumnavigation distance to obtain a ratio. A ratio close to one indicates that the distances to follow a land or water route are similar, and the distance saved by crossing the waterbody is minimal.

#### Tailwind assistance covariate

We used the R package *RNCEP* [21] to retrieve surface-level U (east-west) and V (north-south) wind components from the NCEP/NCAR Reanalysis data provided by the NOAA/OAR/ESRL PSL, Boulder, CO, USA [22, 23]. Wind components for each departure point were interpolated from a global grid with a spatial resolution of 2.5° latitude and 2.5° longitude and a temporal resolution of 6 h (daily at 00, 06, 12, and 18 h UTC), using the function ‘NCEP.interp()’ with the default parameter of linear interpolation. We assigned an optimal direction for each waterbody crossing, based on the observed migratory routes and known breeding destinations of individual birds. The optimal direction for all birds to cross the Caribbean Sea was west (270°), the Gulf of Honduras was northwest (315°), and the optimal direction to cross the Gulf of Mexico varied from northwest (315°), to north (0°), or northeast (45°), for individuals migrating to Texas, Manitoba, or Florida, respectively. We derived the tailwind assistance (m/s) at each departure point from the U and V wind components, using the *RNCEP* function ‘NCEP.Tailwind()’ [21]. We specified optimal direction for each crossing as the assumed preferred flight direction, and did not specify airspeed. The value of the tailwind assistance will be positive when the wind is flowing in the optimal direction of travel, and negative for wind flowing against the optimal direction (i.e. headwind). We scaled and centered the variables of distance savings and tailwind assistance for easier comparison in a linear mixed model using the R function ‘scale()’. We tested the likelihood of crossing over a waterbody in a logistic regression, with tailwind assistance and water:land distance ratio as fixed effects, and individual bird as a random effect to control for repeated measures of individuals that made multiple water crossings.

To visualize the distribution of winds available at departure locations and times compared to the birds’ decisions to cross or detour around waterbodies, we calculated windspeed (m/s) and direction (with 0° as north) from the U and V wind vectors using the R package *rWind* [24]. We plotted windroses [25] of windspeed and direction frequency, and circular histograms of birds’ track bearings. Our sample size for the windrose and histogram for water crossings (*n* = 23) included one additional departure point that was excluded from the model because we did not have a GPS location over land, and therefore could not confidently calculate a distance ratio.

### Statistical analyses

We used R version 4.0.3 for all statistical analyses [26]. We used the Bayesian package *brms* [27] to fit the linear mixed models, which were run with default uninformative priors, four chains, and a minimum of 2000 iterations. We examined model residuals to confirm that variables reasonably met linearity assumptions, and models were validated with posterior predictive checks to ensure complete mixing of chains and that posterior distributions did a good job of predicting new data. We calculated R^2^ for Bayesian models to evaluate the proportion of variance explained by the model terms [28]. All model results reported include 95% Bayesian credible intervals (CI). Maps were created in ArcGIS 10.7 [29].

## Results

**Fig. 1:**
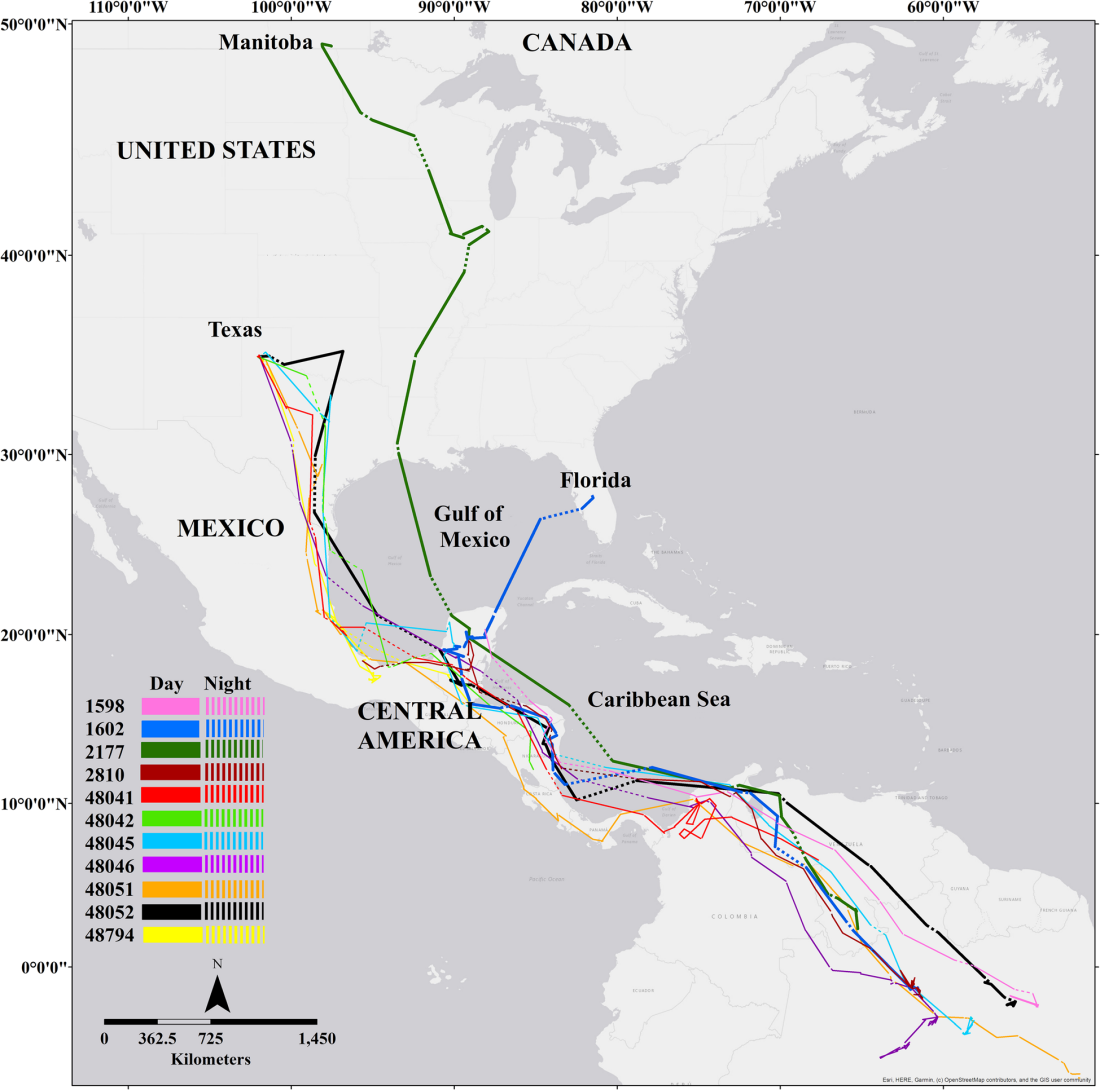
Global Positioning System locations of eleven spring migration tracks of purple martins from 2018 to 2020 during spring migration to three breeding locations (Texas and Florida, USA and Manitoba, Canada) from their South American overwintering grounds. Straight lines connect GPS locations and do not necessarily reflect true migratory paths

**Fig. 2:**
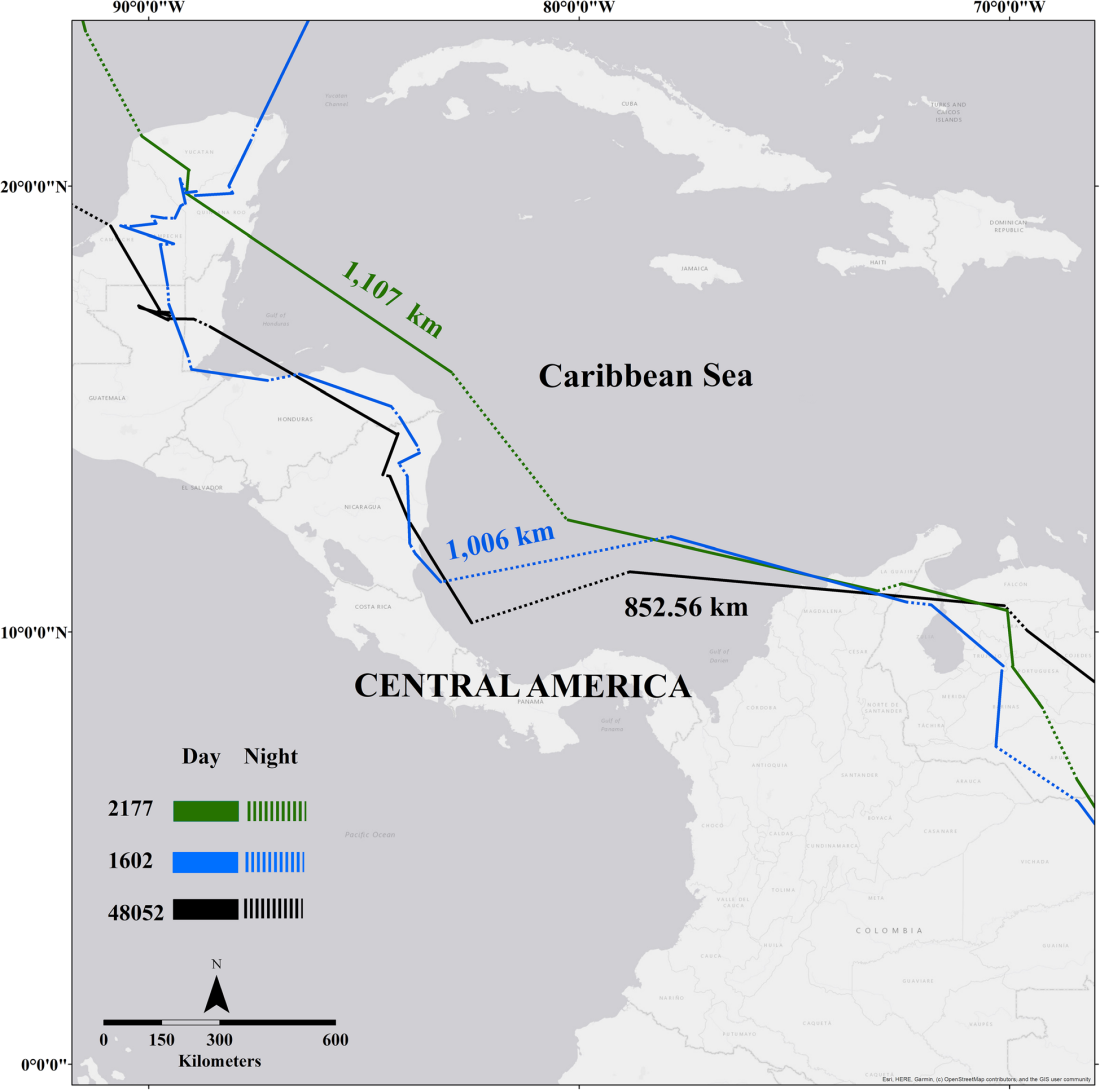
Longest migration distances over a 24-h span crossing the Caribbean Sea for three purple martins returning to their breeding grounds in Manitoba, Canada (2177), Florida (1602) and Texas, USA (48052). Distances represent straight line flight over water and do not necessarily reflect exact flight paths

During the breeding seasons of 2018–2020 we retrieved 12 GPS units (*n* = 2 FL, *n* = 9 TX, *n* = 1 MB). A tag retrieved in Texas recorded only 12 fixes and was excluded from our analysis. Among the remaining 11 tags, two began recording migration en route and two tags stopped recording data before birds reached their breeding sites (Figs. 1, 2). The number of useable points per individual ranged between 45 and 80 fixes per GPS unit (recorded over a sampling period up to ~ 40 days) with a total of 710 points from all 11 retrieved tags. After removing the points recorded outside of the migratory period (at breeding and wintering sites), a total of 461 points were used for further analysis of spring migration. Spring migration routes and timing fell within the range of what had been previously recorded when using light-level geolocator tags for these same breeding populations ([13, 14], Fraser et al. unpublished data). Tag retrieval rates (~ 12%) were lower than previously reported in this species when using the same GPS units (tag retrieval at 17%, [30]), or when using tracking tags of similar weight and dimensions (retrieval rate of geolocators at 21–61%, [30, 31]), and as compared to return rates for birds that were banded only (25–48%, [31]).

We found that 10 of 11 birds made large, open-water crossings during spring migration that included night flight (Table 1). These open water flights occurred when birds crossed the Caribbean Sea (which includes the Gulf of Honduras) or the Gulf of Mexico. Total, straight line distances between GPS locations for these open-water crossings ranged from 96.9–1107 km. The average total distance for night flights over water per individual was 357.11 ± 25.69 km compared to 559.79 ± 49.82 km for flights over water during the day (Fig. 2). The average total spring migration distance for Texas birds was 6526.85 ± 660.98 km and for Florida birds was approximately 5530.53 ± 474.66 km. The total migration distance for the Manitoba bird was 7611.8 km (Table 2). All birds that made some crossing of the Caribbean Sea (*n* = 8) and the Gulf of Mexico (*n* = 8) used night flight. Six birds (55%) that made large crossings of the Caribbean Sea or the Gulf of Mexico initiated these crossings during dark hours, not only at the end to complete the crossings. All 11 birds also made small, overland flights at night that were not associated with barrier crossing.

**Table 2:** Total migration distance for 11 purple martins with the mean and standard errors calculated for the distance travelled over land and water during the day and night

Bird ID	Breeding ground	Total distance (km)	Distance over land/day (km) (mean ± SE)	Distance over land/night (km) (mean ± SE)	Distance over water/day (km) (mean ± SE)	Distance over water/night (km) (mean ± SE)
1598	Florida	4859.26	258.77 ± 34.75	43.71 ± 14.38	592 ± 0.00	362.50 ± 16.62
1602	Florida	6201.80	151.81 ± 28.96	44.90 ± 10.98	452.63 ± 145.31	432.5 ± 119.85
2810	Texas	3907.73	240.28 ± 25.16	45.39 ± 13.08	630 ± 0.00	419 ± 74.95
48041	Texas	7138.05	163.29 ± 26.59	13.30 ± 3.75	353.5 ± 141.77	302 ± 61.51
48042	Texas	3620.68	206.49 ± 37.89	68.50 ± 28.43	635.69 ± 0.00	235 ± 0.00
48045	Texas	8079.28	275.04 ± 45.72	34.12 ± 7.03	672.5 ± 109.95	275.5 ± 59.74
48046	Texas	6784.23	209.21 ± 36.37	22.05 ± 4.37	428 ± 104.19	368.66 ± 52.39
48051	Texas	8397.53	200.92 ± 21.80	37.35 ± 7.69	497 ± 0.00	0.00
48052	Texas	7515.37	235.98 ± 57.95	50.63 ± 19.13	666.33 ± 159.49	440 ± 173.05
48794	Texas	4152.84	187.07 ± 43.88	24.17 ± 5.27	154.00 ± 0.00	417.00 ± 0.00
2177	Manitoba	7611.80	207.98 ± 33.37	53.23 ± 14.42	787.67 ± 8.03	377.50 ± 65.40

Among the birds that migrated to Florida, Texas, and Manitoba, the longest straight-line distance between points covered within 24 h occurred while birds were migrating over the Caribbean Sea. These included a 1222 km total flight with 1006 km occurring over water (bird ID 1602, FL colony), a 1378 km total flight with 852.56 km occurring over water (bird ID 48052, TX colony), and a 1274 km total flight with 1107 km occurring over water (bird ID 2177, MB colony; Fig. 2). The mean flight distance in 24 h was approximately 295.03 ± 17.9 km.

Migration speed over open water was highest over the Caribbean Sea at 79 km/h during the day (tag ID 48052) and 50 km/h at night (tag ID 1602). The highest migration speed during the day over land was 57 km/h (tag ID 48794) and 32 km/h at night (tag ID 1598; Table 3). The total average migration speed was 12 ± 0.70 km/h.

**Table 3:** Average migration speed over land and water (km/h) during the day and night as well as highest migration speed over land and water during the day and night (km/h). C: over Caribbean Sea, G: over Gulf of Mexico

ID	Breeding ground	Migration speed land/day (mean ± SE)	Migration speed land/night (mean ± SE)	Migration speed water/day (mean ± SE)	Migration speed water/night (mean ± SE)	Highest migration speed land/day	Highest migration speed land/night	Highest migration speed water/day	Highest migration speed water/night
1598	Florida	22 ± 5	4 ± 4	50 ± 0	30 ± 1	37	32	49 C	28 C
1602	Florida	13 ± 2	4 ± 1	38 ± 12	36. ± 1	45	21	53 G	50 C
2810	Texas	21 ± 2	4 ± 1	53 ± 0	35 ± 6	31	14	53 C	43 C
48041	Texas	14 ± 2	1 ± 0	30 ± 12	2 ± 5	53	9	46 C	32 G
48042	Texas	17 ± 3	6 ± 2	53 ± 0	20 ± 0	42	26	53 G	19 G
48045	Texas	23 ± 4	3 ± 1	56 ± 9	23 ± 5	57	11	69 C	30 C
48046	Texas	17 ± 3	2 ± 0	36 ± 9	31 ± 4	42	7	57 G	41 C
48051	Texas	17 ± 25	3 ± 1	41 ± 0	0	34	18	41 C	0
48052	Texas	20 ± 5	4 ± 1	56 ± 13	37 ± 1	57	28	80 C	38 G
48794	Texas	16 ± 4	2 ± 4	13 ± 0	35 ± 0	57	6	29 G	19 G
2177	Manitoba	17 ± 3	4 ± 1	66 ± 1	31 ± 5	43	17	67 C	39 C

During predominantly daytime flights, an increase in available daylight did not influence distances (log km) traveled (slope 0.0024 [− 0.0017, 0.0067]; Supplemental Fig. S1a), but there was a statistically significant positive effect during predominantly nighttime flights (slope 0.013 [0.0065, 0.019]; Supplemental Fig. S1b). The amount of daylight that occurred within the nighttime flight segments ranged from four to 176 min (mean 67 min, SD 40), and the distance flown during the nighttime flights ranged from zero to 594 km (mean 68 km, SD 110). Within the nighttime flight segments, a 50% increase in amount of daylight would correspond to a predicted mean 0.5% increase in distance traveled.

**Fig. 3:**
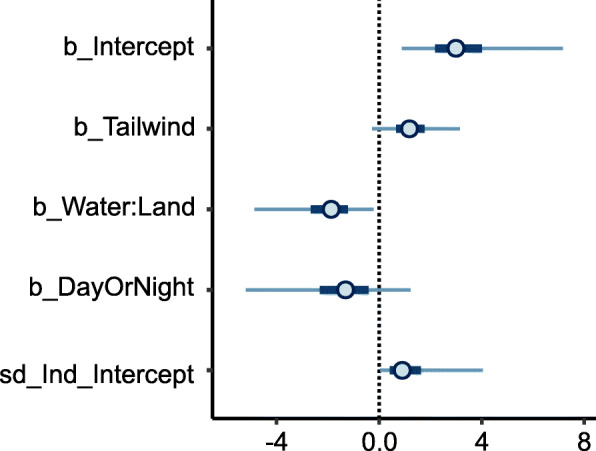
Bayesian linear mixed model coefficients for the effects of tailwind assistance, water:land distance ratio, and time of day on purple martin water crossings. Only the distance ratio had a significant effect on decisions to initiate water crossings (they were less likely to cross a water body as the distance savings decreased). Coefficient estimates are shown with 50 and 95% credible intervals. Continuous variables were scaled and centered prior to analysis

**Fig. 4:**
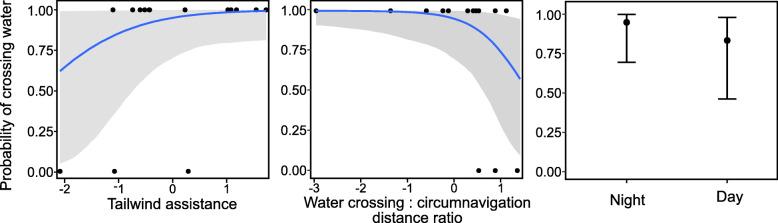
The predicted probability of purple martins initiating a water crossing, rather than detouring to circumnavigate over land, does not increase significantly with an increase in tailwind assistance in the optimal direction of travel (left), or during night vs day (right). Probability of crossing decreases significantly as the water to land ratio increases, and the potential savings in distance therefore decrease (center). Shading represents 95% Bayesian credible intervals. Continuous variables were scaled and centered prior to analysis

**Fig. 5:**
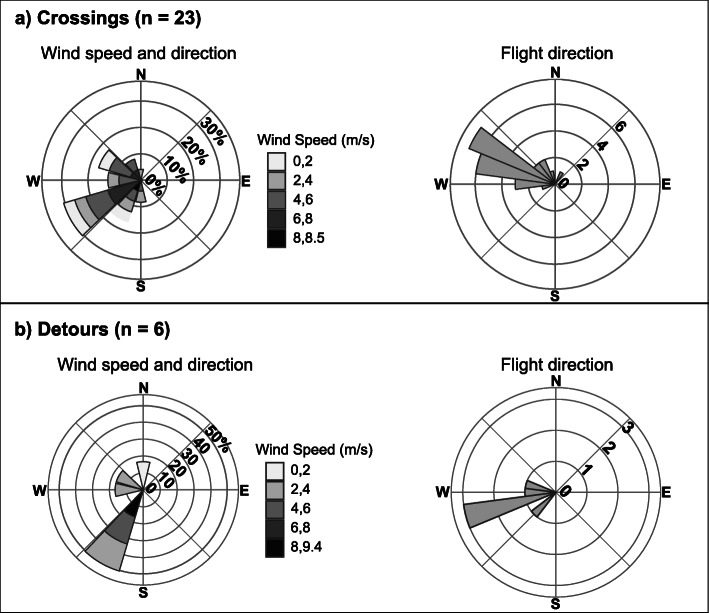
Windroses of the frequency of wind speeds at departure points for purple martins that either crossed (**a**) or detoured (**b**) around waterbodies (left in each panel). Corresponding histograms of flight directions from GPS tracked individuals (right in each panel). Bars on all plots indicate the directions that wind or flights are moving toward: winds during both crossings and detours flowed predominantly toward the southwest, and bird flights were directed predominantly toward the northwest (crossings) and west-southwest (detours)

The probability of purple martins initiating a water crossing, rather than detouring to circumnavigate over land, decreased as the water to land ratio increased, and the potential distance savings thus decreased (Table 4, Figs. 3, 4). That is, when the distances of a water crossing and the alternative circumnavigation were similar, birds were more likely to take the overland route than the overwater route. Seven of the 22 water crossings, and five of the six detours, were initiated during night, and we did not detect an effect of time of day (day versus night) on the decision to initiate water crossings. The Bayes R^2^ for this model was 0.30 (Estimated Error: 0.10; CI = 0.10–0.50). Winds at the departure points and times were predominantly to the southwest for both water crossings and detours, whereas the birds’ flight directions were predominantly to the northwest during crossings and west-southwest for detours (Fig. 5).

**Table 4:** Logistic regression results for covariates predicting whether migrating purple martins will cross, or detour around, waterbodies. Tailwind is the wind assistance (m/s) in the optimal direction of travel; Water:Land refers to distance saved by crossing a waterbody (higher ratio = less distance saved), and DayOrNight is time of day that flights were initiated. CI = lower and upper Bayesian credible intervals. Continuous variables were scaled and centered prior to analysis

Covariate	Estimate	Est.Error	l-95% CI	u-95% CI	Rhat	Bulk_ESS	Tail_ESS
Intercept	3.24	1.57	0.88	7.16	1.00	2538	1744
Tailwind	1.25	0.86	−0.28	3.15	1.00	5795	4345
Water:Land	−2.04	1.18	−4.86	−0.23	1.00	3319	1971
DayOrNight	−1.47	1.62	−5.20	1.22	1.00	3213	1871

## Discussion

Our study provides the first tracking evidence that swallows, generally considered to be diurnal migrants, can also incorporate night flights into their migrations, particularly when crossing large, open-water barriers. The use of night flights in species usually characterized as diurnal, fly-and-forage, migrants was predicted to occur at areas with reduced foraging opportunities, such as at barrier crossing [4]. This has recently been shown in falcons [11], but had not been demonstrated for diurnal songbirds such as swallows, that commonly cross ecological barriers [13, 14] where some night flight was also predicted to be required to cross such great distances. Our migration-tracking of purple martins using high precision GPS units support hypotheses for the use of nocturnal flights at barrier crossing by otherwise diurnal migrants [4] and builds upon other recent migration-tracking evidence demonstrating the combination of day and night flights in long-distance migration (e.g. [32]). Our results are consistent with the notion that these primarily diurnal birds use night flight, particularly when making large open-water crossings, to help achieve time- and energy-savings when foraging conditions are suboptimal.

Ten of the eleven purple martins that we tracked used night flight when crossing over water. In some ways this would be expected as birds crossing open water at distances greater than 1000 km would not have anywhere to stop for rest, and with flight speeds of 19–36 km/h, could not complete the crossing during 12 h of daylight [33]. Large barrier crossings may generally require that chiefly nocturnal or diurnal migrants include flights during light conditions when they do not typically migrate. Despite a high prevalence of nocturnal flight in our study, the average migration distance over water was greater during the day as compared to the night. In part, this may be due to night flight being used primarily to finish off the crossing of ecological barriers (12 of 19 of the total number of open-water flights at night were to complete crossings). Similar to our findings for flights extending to cross day-night boundaries, several species of songbird incorporate some daytime flight into their otherwise nocturnal migrations in order to finish the crossing of the expansive migration barrier of the Sahara Desert [3, 5, 34].

However, we also found that 32% of all open water crossings were initiated at night. Night flights were therefore not only used to complete crossings initiated during daytime as could be predicted based upon distances and expected rates of travel for diurnal migrants crossing ecological barriers [9, 16]. Our results support optimal timing hypotheses for fly-and-forage migrants, where it is predicted that daily travel schedules may shift toward night flights in areas that do not offer good foraging opportunities [4]. Ocean crossing during dark hours may confer time- or energy-savings while supporting an otherwise diurnal, fly-and-forage, migratory strategy in songbirds. Aerial insectivores such as purple martins and other swallows may be able to catch insect prey while heading in a general migratory direction during the day or make short stops or detours to accomplish this task while maintaining their migration [9]. Or, migratory flights may be undertaken in the morning daylight hours, leaving the afternoon for foraging and refueling [4]. Indeed, such a strategy may be evident in bank swallows (*Riparia riparia*), where during fall migration travel speeds were slower, suggesting they were actively refueling while migrating [35]. However, the degree to which other swallow species use a fly-and-forage strategy, and the conditions that promote its use, require further investigation using migration tracking.

During open-ocean or other barrier crossing, the advantages of being able to forage while migrating may be reduced or eliminated, as aerial insect availability may be limited or absent over open ocean [4]. Birds that engage in ocean crossing and incorporate night flights may reflect a time- and/or energy-minimization strategy [36, 37], where barrier crossing at the Caribbean Sea or Gulf of Mexico may greatly reduce overall distances travelled, and thus minimize the overall time and energy required to complete spring migration [35, 36]. Indeed, the ocean crossings we documented reflect significant ‘short cuts’, as compared to an overland route throughout the same regions (e.g. Gulf of Mexico crossing 871 km versus around at 937 km).

In addition to large, open-water flights at night, we observed shorter night flights over land. Average night flights over land were also much shorter than average daytime flights over land. Like night flights over water where foraging opportunities are low, night flights over land may also occur over areas that offer limited foraging opportunities [4]. However, some of the flights we documented as short ‘night’ flights, occurred around sunrise or sunset and therefore could have been completed in the twilight hours. A positive relationship between ‘nighttime’ flight distances and amount of daylight available during each 12 h track segment (Supplemental Figure S1b) indicates that some ‘night’ flights likely took place during daylight hours near sunrise or sunset. Indeed, four swallow species that utilize a fly-and-forage strategy migrated during twilight as revealed through automated radio telemetry [12].

Our data indicate that the probability that purple martins will initiate water crossings during northbound spring migration does not increase significantly with stronger winds flowing in a direction that would support travel in optimal directions (Table 4, Figs. 3, 4). However, wind measured on the days that martins navigated waterbodies, whether by crossing or detouring, were predominantly to the southwest, which would not confer an advantage to birds traveling predominantly to the northwest (Fig. 5). These results align with some previous observations for night-migrating passerines, where there was little selection for facilitating winds and individuals continued directional migration flights despite variation in wind direction and strength [38].

Variation in survival rates between years and sites could have contributed to the variation in tag retrieval within our study and the lower tag retrieval rate overall. Generally, tagged birds in this species tend to have similar return rates to birds that were banded only [30] and different tag types deployed in the same year at the same sites had comparable return rates [29]. However, since this study was not designed to test for factors contributing to tag retrieval rate, we cannot rule out a potential tag effect on survival. Further, sampling and re-sighting methods were not necessarily consistent across sites within this study or as compared to earlier studies, e.g., re-sighting, re-capture, and initial tagging occurred at varying times within the nesting cycle at different sites which could have contributed to variation in tag retrieval rates. A future study aimed specifically at investigating factors that may contribute to the retrieval rates of GPS units could better identify factors that influence retrieval rates.

## Conclusion

This is the first study of a swallow to examine circadian patterns of flight across migration to see how selection has potentially shaped day-night circadian migratory behaviours. Our study demonstrates that a species generally considered to be a diurnal migrant incorporates a large amount of nocturnal flight into its migrations, particularly at barrier crossing. Night flights in an otherwise diurnal species, may be favoured where foraging opportunities are low and to contribute to a time- and energy-saving strategy. Future research could further target within-day patterns of movement, to test hypotheses for how diel foraging and refueling patterns may support the combination of night and day flights and the advantages of nocturnal open-water crossing in otherwise diurnal migrants.

## Supplementary Information

**Additional file 1: Table A.** Four breeding colonies of purple martins (*Progne subis*) along with the number of GPS units (Lotek) deployed and retrieved at each site for spring migration tracking (2017–2020). **Figure S1.** Relationship between distance traveled by Purple Martins and amount of daylight available in each 12 h tracking period. Daylight hours are defined as the time between sunrise and sunset, and the amount of daylight per track was calculated according to the GPS locations and fix times at the beginning and end of a track (i.e. time between sunrise/sunset at the bird’s start location and sunset/sunrise at the bird’s end location). During predominantly daytime flights, an increase in available daylight did not influence distances traveled (a), but there was a statistically significant effect during predominantly nighttime flights (b). These results indicate that some of the flight attributed to nighttime likely occurs during daylight hours.

## Data Availability

Data will be made publicly available.
