# Supplementary material for: The use of nocturnal flights for barrier crossing in a diurnally migrating songbird
Source: Mov Ecol. 2021 Apr 26;9:21. doi: 10.1186/s40462-021-00257-7 (PMC8073915; doi:10.1186/s40462-021-00257-7)
Supplement: Supplementary file 1 — Additional file 1: Table A. Four breeding colonies of purple martins (Progne subis) along with the number of GPS units (Lotek) deployed and retrieved at each site for spring migration tracking (2017–2020). Figure S1. Relationship between distance traveled by Purple Martins and amount of daylight available in each 12 h tracking period. Daylight hours are defined as the time between sunrise and sunset, and the amount of daylight per track was calculated according to the GPS locations and fix times at the beginning and end of a track (i.e. time between sunrise/sunset at the bird’s start location and sunset/sunrise at the bird’s end location). During predominantly daytime flights, an increase in available daylight did not influence distances traveled (a), but there was a statistically significant effect during predominantly nighttime flights (b). These results indicate that some of the flight attributed to nighttime likely occurs during daylight hours. [file 40462_2021_257_MOESM1_ESM.docx]

**Supplemental material**

**Table A.** Four breeding colonies of Purple martins (*Progne subis*) along with the number of GPS units (Lotek) deployed and retrieved at each site for spring migration tracking (2017-2020).

| State/Province | Latitude | Longitude | # Deployed | # Retrieved |
| --- | --- | --- | --- | --- |
| Florida | 28°21’34.92” N | 81°35’28.68” W | 10 | 2 |
| Pennsylvania | 42°11’48.98” N | 80°14’53.59” W | 10 | 0 |
| Texas | 35°02’23.01” N | 101°56’00.41” W | 62 | 9 |
| Manitoba | 49°44’03.34’’ N | 97°07’53.00’’ W | 16 | 1 |


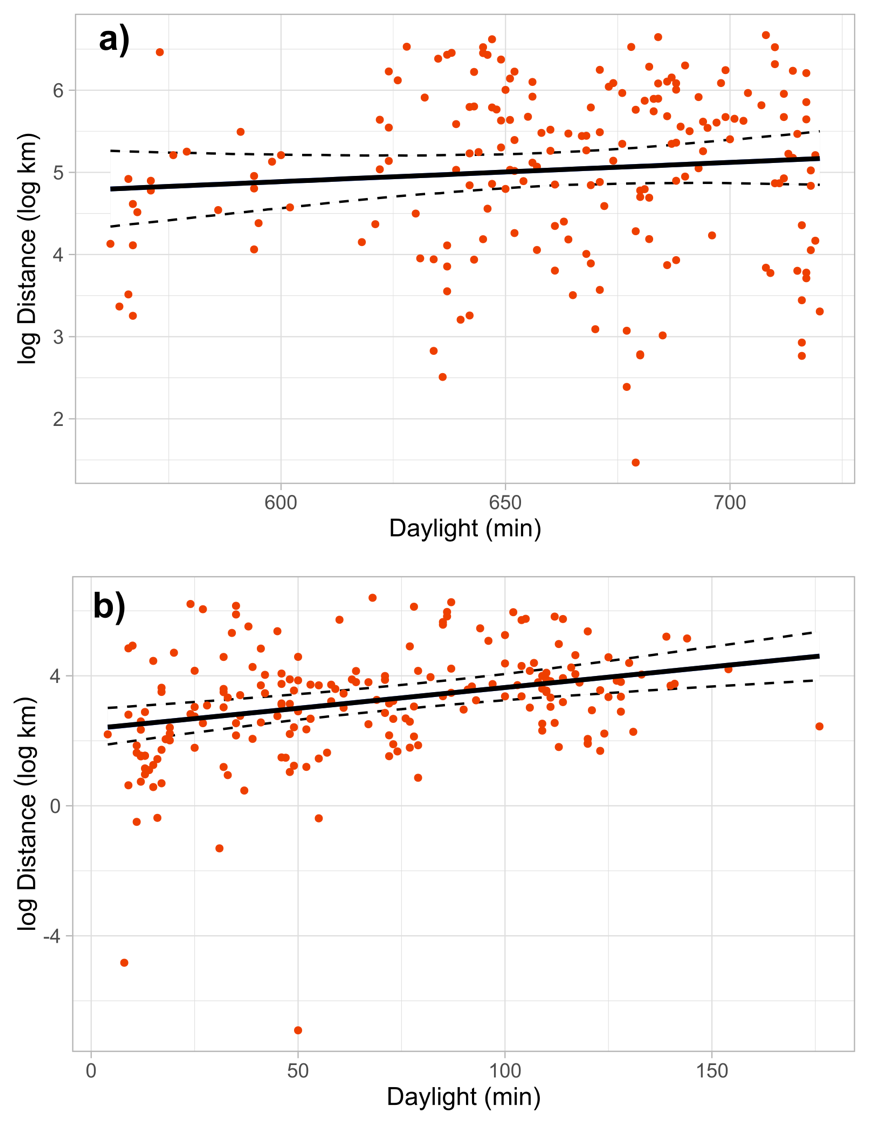


**Figure S1.** Relationship between distance traveled by Purple Martins and amount of daylight available in each 12-hour tracking period. Daylight hours are defined as the time between sunrise and sunset, and the amount of daylight per track was calculated according to the GPS locations and fix times at the beginning and end of a track (i.e. time between sunrise/sunset at the bird’s start location and sunset/sunrise at the bird’s end location). During predominantly daytime flights, an increase in available daylight did not influence distances traveled (a), but there was a statistically significant effect during predominantly nighttime flights (b). These results indicate that some of the flight attributed to nighttime likely occurs during daylight hours
